# Occurrence and Effects on Weaning From Mechanical Ventilation of Intensive Care Unit Acquired and Diaphragm Weakness: A Pilot Study

**DOI:** 10.3389/fmed.2022.930262

**Published:** 2022-07-22

**Authors:** Michele Bertoni, Simone Piva, Alessandra Beretta, Federica Bongiovanni, Riccardo Contarino, Ricard Mellado Artigas, Lucia Ceresoli, Mattia Marchesi, Michele Falappi, Marta Belleri, Alberto Goffi, Matteo Pozzi, Frank Antonio Rasulo, Nicola Latronico

**Affiliations:** ^1^Department of Anesthesia, Critical Care and Emergency, Spedali Civili University Hospital, Brescia, Italy; ^2^Department of Medical and Surgical Specialties, Radiological Sciences and Public Health, University of Brescia, Brescia, Italy; ^3^Department of Anesthesiology, Surgical ICU, Hospital Clinic de Barcelona, Barcelona, Spain; ^4^CIBER de Enfermedades Respiratorias, Instituto de Salud Carlos III, Madrid, Spain; ^5^Department of Medicine and Interdepartmental Division of Critical Care Medicine, University of Toronto, Toronto, ON, Canada; ^6^Department of Emergency and Intensive Care, ASST Monza, Monza, Italy

**Keywords:** intensive care unit-acquired weakness, diaphragmatic weakness, simplified peroneal nerve test, thickening fraction of the diaphragm, maximum inspiratory pressure, coexistence, weaning from mechanical ventilation

## Abstract

**Purpose:**

Limb intensive care unit (ICU)-acquired weakness (ICUAW) and ICU acquired diaphragm weakness (DW) occur frequently in mechanically ventilated (MV) patients; their coexistence in cooperative and uncooperative patients is unknown. This study was designed to (1) describe the co-occurrence of the two conditions (2) evaluate the impact of ICUAW and DW on the ventilator-free days (VFDs) at 28 days and weaning success, and (3) assess the correlation between maximal inspiratory pressure (MIP) and thickening fraction (TFdi) in patients with DW.

**Methods:**

This prospective pilot study was conducted in a single-center on 73 critically ill MV patients. Muscle weakness was defined as a Medical Research Council score < 48 in cooperative patients or a bilateral mean simplified peroneal nerve test < 5.26 mV in uncooperative patients. Diaphragm dysfunction was defined as MIP < 30 cm H_2_O or as a TFdi < 29%. Weaning success was defined according to weaning according to a new definition (WIND).

**Results:**

Fifty-seven patients (78%) had ICUAW and 59 (81%) had DW. The coexistence of the two conditions occurred in 48 patients (65%), without association (χ^2^ = 1.06, *p* = 0.304). In the adjusted analysis, ICUAW was independently related to VFDs at 28-days (estimate difference 6 days, *p* = 0.016), and WIND (OR of 3.62 for having WIND different than short weaning), whereas DW was not. The linear mixed model showed a significant but weak correlation between MIP and TFdi (*p* < 0.001).

**Conclusion:**

This pilot study is the first to explore the coexistence of ICUAW and DW in both cooperative and uncooperative patients; a lack of association was found between DW and ICUAW when considering both cooperative and uncooperative patients. We found a strong correlation between ICUAW but not DW with the VFDs at 28 days and weaning success. A future larger study is warranted in order to confirm our results, and should also investigate the use of transdiaphragmatic twitch pressure measurement during bilateral anterior magnetic phrenic nerve stimulation for the diagnosis of DW.

## Introduction

Limb intensive care unit-acquired weakness (ICUAW), a common consequence of critical illness, is defined as a clinically detected weakness in critically ill patients in whom there is no plausible etiology other than critical illness (1). ICUAW can persist for months after ICU discharge, increasing the incidence of physical and psychological sequelae and resulting in poor long-term functional status and quality of life. Muscle weakness might be present in up to 80% of critically ill mechanically ventilated (MV) patients (2). When the weakness involves the diaphragm, the term used is ICU Acquired Diaphragmatic Weakness (DW). DW is associated with difficult respiratory weaning, and increased morbidity and mortality; its prevalence can be as high as 63–80% during the ICU stay (3).

There is some debate as to whether ICUAW and DW represent two different epiphenomena of the same pathological condition or different diseases (4–6). Studies on this topic are controversial, mainly because the coexistence of these two conditions has been assessed using different diagnostic approaches (7). The diagnosis of ICUAW relies on the Medical Research Council (MRCss) scale, a clinical scale that requires full patient cooperation (4). In uncooperative patients, the function of peripheral nerves and muscles can be assessed using appropriate electrophysiological investigations of peripheral nerves and muscles, which require specialized personnel and are time-consuming (8, 9). The gold standard of DW diagnosis is based on the measurement of transdiaphragmatic twitch pressure (PdiTw) generated in response to bilateral anterior magnetic phrenic nerve stimulation (BAMPS). Alternative methods are the thickening fraction of the diaphragm (TFdi) obtained with ultrasound, or maximum inspiratory pressure (MIP) generated during a prolonged expiratory occlusion maneuver (10–12). To date, there are no studies assessing the coexistence of ICUAW and DW using electrophysiological tests for ICUAW and MIP and/or TFdi for DW, in both cooperative and uncooperative critically ill MV patients. The two largest studies on this topic included only cooperative patients and used MRCss to diagnose ICUAW (4, 13).

Therefore, the primary objective of this pilot study was to measure the coexistence of ICUAW and DW using objective techniques in both cooperative and uncooperative patients. In addition, we also investigated the association between ICUAW and DW with ventilator-free days (VFDs) at 28 days and weaning success. Finally, we assessed the correlation between MIP and TFdi.

## Materials and Methods

### Patient Selection

This prospective pilot study was conducted between May 2019 and January 2021 at the Spedali Civili University affiliated Hospital of Brescia, Italy. The study was approved by the local Ethical Committee and informed consent was obtained for all patients. The STROBE guidelines for reporting observational studies were followed (14).

All consecutive patients admitted to the ICU for at least 72 h and who were MV on assisted mechanical ventilation for at least 48 h, were assessed for enrolment. Exclusion criteria were: age < 18 years old; pre-existing neuromuscular disorders that may have affected the diagnosis of ICUAW and DW; impossibility to assess the presence of ICUAW neither with MRCss nor with simplified peroneal nerve test (PENT). We recorded demographic data, ICU admission severity scores (SAPS II and admission SOFA), comorbidities, the reason for ICU admission, ICU and hospital length of stay (LOS), VFDs at 28 days, weaning success, and hospital mortality. Weaning success was assessed using the Weaning according to a New Definition (WIND) classification. Briefly, patients were categorized into four groups according to the time of weaning: patients in whom no separation attempt was made (Group: NW); patients who terminated the weaning process within 1 day from the first attempt (Group 1: short weaning); patients who completed the weaning process after 1 day but within 1 week from the first Spontaneous breathing trials (SBT; Group 2: difficult weaning); patients who required more than 7 days to be separated from the ventilator with success (Group 3a: prolonged weaning); patients who required more than 7 days to be separated from the ventilator without success (Group 3b: weaning failure; 15). Successful weaning was defined as separation from the MV for at least 7 days.

### Study Protocol

**FIGURE 1:**
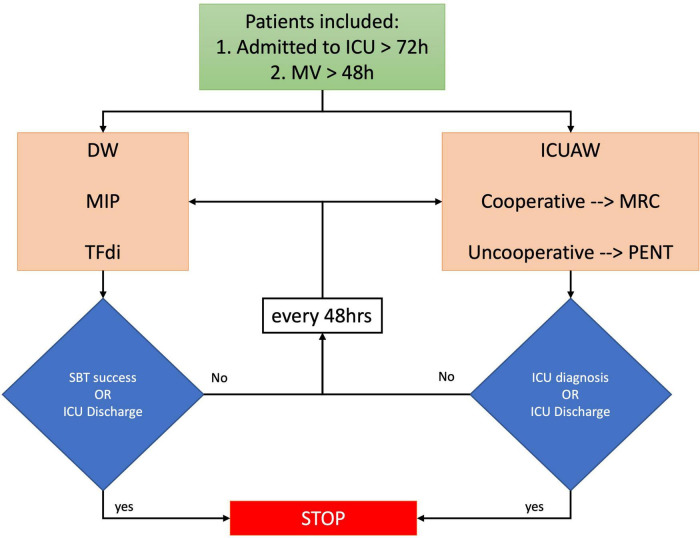
Study protocol. Patients admitted to intensive care medicine (ICU) for >72 h and mechanically ventilated for >48 h were screened. ICUAW was evaluated using MRC and PENT, and DW was diagnosed with MIP and TFdi. MV, mechanical ventilation; TFdi, diaphragm thickening fraction; MIP, maximal inspiratory pressure; MRC, medical research council scale; PENT, simplified peroneal nerve test; SBT, spontaneous breathing trial (PSV 6/6 cmH2O).

Data collection started on the first day of *assisted mechanical ventilation*. The presence of ICUAW was monitored every 48 h until an ICUAW diagnosis was made or until the patient was discharged from ICU. Data on DW were collected every 48 h until SBT success or ICU discharge. All patients were ventilated in pressure support ventilation (PSV). MIP measurements were performed only when P0.1 (airway occlusion pressure at 100 ms) ranged between 1.5 and 3 cm H_2_O, to avoid over-or under-estimation (12). Measurements were repeated until the day of separation from the MV. SBT were performed in PSV using an inspiratory pressure of 6 cm H_2_O and a PEEP level of 6 cm H_2_O (16). The study protocol is represented in Figure 1.

### Muscles Assessment

The presence of ICUAW was monitored every 48 h either with MRCss (cooperative patients) or with bilateral PENT performed at the bedside (uncooperative patients). We validated PENT as a screening tool for ICUAW in two previous studies (8, 9), and we found a threshold value of 5.26 mV with a sensitivity of 100% and specificity of 85%. In the case of pathological MRCss, PENT was performed to confirm the diagnosis; when ICUAW was excluded by MRCss, PENT was not performed.

### Diaphragm Assessment

Maximum inspiratory pressure values were obtained by averaging the results of three expiratory occlusion maneuvers. In cooperative patients, the MIP was obtained by asking the patient to voluntarily perform a maximal inspiratory effort starting from the functional residual capacity (10). In uncooperative patients, the maximal pressure generated against a prolonged expiratory occlusion maneuver was considered MIP. The respiratory drive was intensified by inducing an airway occlusion for a 25 to 30 s period, in this manner eliciting a maximal inspiratory effort (10, 12). To minimize bias resulting from sedation and low respiratory drive, we performed MIP only when P0.1 ranged between 1.5 and 3 cm H_2_O during the breath just before the occlusion. As previously reported, the respiratory drive should not affect MIP estimation at these P 0.1 values (12, 17). MIP was always directly measured from the ventilator’s airway pressure waveforms using either Maquet Servo U (Getinge, Göteborg, Sweden) or Bellavista, 1000e (Imtmedical ag, Buchs, Switzerland) ventilators.

Right hemidiaphragm TFdi was obtained as described elsewhere (18). Briefly, the thickening fraction was computed as the percentage change in thickness between End-Expiration (i.e., minimum muscle thickness, TEE) and Peak Inspiration (i.e., maximal muscle thickness, TPI) visualized in M-mode (TFdi: TPI- TEE/TEE). TFdi was measured while the patients were ventilated in pressure support set to provide an adequate tidal volume and respiratory drive. According to previous studies DW was defined as a MIP less than –30 cm H_2_O or a TFdi < 29% (19).

### Statistical Analysis

Variables are reported as means (SDs), medians (IQRs), or numbers (percentages) as appropriate. The Shapiro–Wilk test was used to assess whether continuous data were normally distributed. The association between DW and ICUAW was evaluated using the χ^2^ test. We coded VFDs at 28 days = 0 if either the subject died within 28 days of mechanical ventilation or if MV for more than 28 days; in all the other cases VFDs at 28 days were defined as 28-days of mechanical ventilation (20).

Logistic regression was used to compare ICU admission baseline characteristics between patients with and without ICUAW or DW. An unadjusted multivariate linear regression or ordered logistic regression was used to determine the association between VFDs at 28 days and weaning success (using WIND as ordered dependent variable), respectively, including the presence of ICUAW and DW as an independent variable, adjusting for the following confounders: age, sex, BMI, and SAPS II. Adjusted logistic regression was then performed keeping all the variables in the model after the selection of confounders by using direct acyclic graphs, Supplementary Figure 1. We did not include SOFA at ICU admission since is strongly correlated to SAPS II. Finally, a linear mixed model was used to correlate MIP and TFdi, using the subject as a random effect, to overcome the issue of the repeated measures. Since this is a pilot study sample size was not calculated. All tests were two-sided, and a *p*-value less than 0.05 was considered statistically significant. No data imputation was performed for these data. All analyses were conducted using R (version 4.1.1).

## Results

During the study period, 73 patients were enrolled. The Median [IQR] age was 65 [16.5] years; 45 (62%) patients had COVID-19 related Acute Respiratory Distress Syndrome (CARDS). Median [IQR] SAPS II was 32 [18], and the median [IQR] admission ICU SOFA score was 4 [3]. The Median [IQR] duration of MV was 10 [10] days; 4 (6%) patients never received any separation attempt (WIND = NW), 11 (15%) patients failed weaning from mechanical ventilation despite separation attempts (WIND 3b), and 58 (79%) patients were weaned (WIND 1–3a), Table 1.

**TABLE 1:** Demographics and outcomes characteristics.

Presence of DW, N°(%)	No ICUAW (*N* = 16; 21%)		ICUAW (*N* = 57; 78%)		Total *N* = 73
	No DW *N* = 5 (6%)	DW *N* = 11 (15%)	No DW *N* = 9 (12%)	DW *N* = 48 (65%)	
Age (years), Median [IQR]	71 [16]	68 [8]	71 [20]	62 [15]	65 [16]
Gender (Female), N° (%)	0 (0%)	5 (46%)	4 (44%)	17 (35%)	26 (36%)
Body mass index (kg/m^2^), Median [IQR]	28.6 [7.8]	24.1 [4.6]	23.1 [8.0]	27.8 [7.6]	27.8 [8.1]
Admission diagnosis, N° (%)					
Polytrauma	0 (0%)	1 (9%)	1 (11%)	2 (4%)	4 (5%)
Respiratory failure (NON-COVID)	1 (20%)	0 (0%)	2 (22%)	5 (10%)	8 (11%)
Sepsis	0 (0%)	0 (0%)	0 (0%)	7 (14%)	7 (9%)
Neurological disease	0 (0%)	0 (0%)	0 (0%)	1 (2%)	1 (1%)
CARDS	4 (80%)	9 (81%)	3 (33%)	29 (60%)	45 (61%)
Cardiac disease	0 (0%)	0 (0%)	0 (0%)	2 (4%)	2 (2%)
Other	0 (0%)	1 (9%)	1 (11%)	0 (0%)	2 (2%)
**Comorbidities, N° (%)**					
0	2 (40%)	3 (27%)	2 (22%)	3 (6.3%)	10 (13%)
1	1 (20%)	3 (27%)	1 (11%)	15 (31%)	20 (27%)
2	2 (40%)	2 (18%)	1 (11%)	12 (25%)	17 (23%)
≥3	0 (0%)	3 (27%)	5 (55%)	18 (37%)	26 (35%)
SAPS II, Median [IQR]	32 [9]	23 [4]	34 [10]	32 [21]	32 [18]
SOFA Score, Median [IQR]	4 [1]	4 [1]	5 [3]	4 [5]	4 [3]
MV duration (days), Median [IQR]	4 [6]	7 [3]	17 [10]	12 [11]	10 [10]
**WIND, N° (%)**					
Group NW: no separation attempt	0 (0%)	1 (9%)	1 (11%)	2 (4%)	4 (5%)
Group 1: short weaning	2 (40%)	8 (72%)	2 (22%)	12 (25%)	24 (32%)
Group 2: difficult weaning	2 (40%)	2 (18%)	3 (33%)	12 (25%)	19 (26%)
Group 3a: prolonged weaning	0 (0%)	0 (0%)	2 (22%)	13 (27%)	15 (20%)
Group 3b: weaning failure	1 (20%)	0 (0%)	1 (11%)	9 (18%)	11 (15%)
ICU LOS (days), Median [IQR]	6 [6]	10 [5]	18 [6]	15 [12]	13 [11]
Hospital LOS (days), Median [IQR]	20 [20]	27 [11]	21 [17]	27 [13]	27.0 [15]
Alive (at Hospital discharge), N° (%)	4 (80%)	9 (81%)	7 (77%)	42 (87%)	62 (84%)

*MV duration, duration of mechanical ventilation; CARDS, COVID-19 ARDS; WIND, Weaning according to a New Definition; ICU LOS, ICU lenght of stay; and Hospital LOS, Hospital lenght of stay.*

**FIGURE 2:**
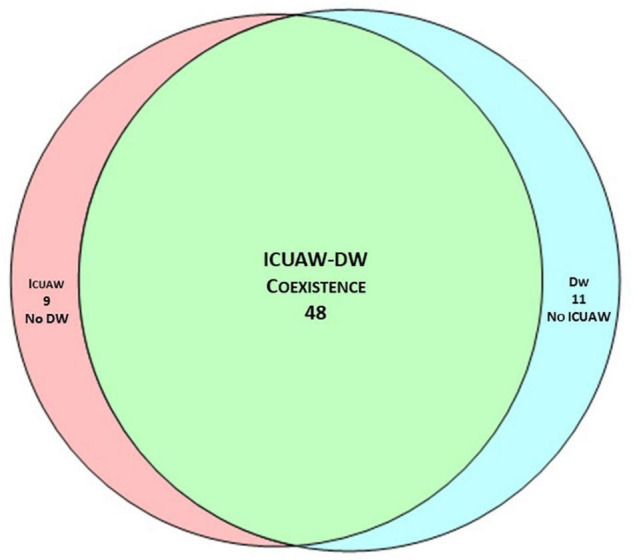
Venn diagram of ICUAW and DW coexistence. The coexistence of the two conditions occurred in 48 patients (65%); there was no association between ICUAW and DW (χ^2^ = 1.06, *p* = 0.304).

Fifty-seven patients (78%) had ICUAW and 59 (81%) had DW. The coexistence of the two conditions occurred in 48 patients (65%), Figure 2. There was no association between the two conditions (χ^2^ = 1.06, *p* = 0.304). Patients with ICUAW had (1) higher SOFA score (median [IQR] 5 [5] vs 4 [1.25], *p* = 0.032), (2) higher SAPS II (24 [10] vs 33 [19], *p* = 0.021), (3) longer duration of MV (median [IQR] 12 [12] vs 6.5 [3.25], *p* = 0.002), (4) more frequently a prolonged weaning (WIND 3a) or a higher percentage of weaning failure despite weaning attempt (WIND 3b), and (5) longer ICU LOS (median [IQR] 15 [11] vs 8 [5], *p* = 0.002), Supplementary Table 1. Conversely, in our cohort, we did not observe an association between DW and ICU admission severity scores, VFDs at 28 days or weaning success (Supplementary Table 2).

In the unadjusted analysis, the presence of ICUAW, SAPS II and SOFA were related to VFDs at 28 days and WIND (using short weaning as reference), Supplementary Tables 3, 4. In the adjusted analysis, patients with ICUAW had fewer VFDs at 28 days (estimate difference 7 days, *p* = 0.016), and had an OR of 3.62 (*p* = 0.050) for WIND different than short weaning (difficult weaning, prolonged weaning or no weaning) compared to patients without ICUAW, Table 2. The presence of DW was not related to either VFD at 28 days or weaning success, Table 2.

**TABLE 2:** Adjusted analysis for mechanical ventilation duration and weaning success as measured by WIND.

*Predictors*	VFDs at 28-days			Wind		
	*Estimates*	*CI*	*p*	*OR*	*CI*	p*
Presence of ICUAW	–6.83	−12.34 – −1.31	0.016	3.62	1.06–13.84	0.050
Presence of DW	4.76	–0.82–10.35	0.093	0.87	0.29–2.68	0.814
SAPS II	–0.27	–0.52 – −0.02	0.034	1.10	1.04–1.16	0.001
Age (years)	–0.14	–0.31–0.04	0.133	1.03	0.99–1.07	0.115
Gender, Male	–0.43	–5.32–4.47	0.862	2.39	0.86–7.01	0.106
Body mass index	–0.22	–0.63–0.20	0.296	1.01	0.92–1.11	0.804

*MV duration, duration of mechanical ventilation; WIND, Weaning according to a New Definition; and CI, 95% Confidence Interval.*

*p is calculated using WIND = Group 1: short weaning is used as reference class.

**FIGURE 3:**
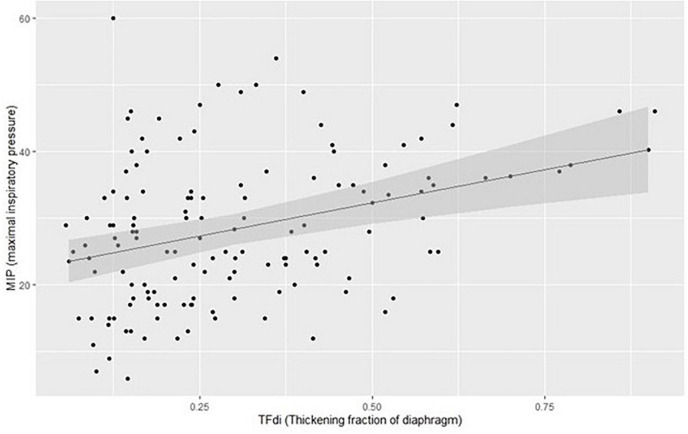
Plotting of model estimates of the correlation between MIP (maximal inspiratory pressure) and TFdi (thickening fraction of diaphragm).

The linear mixed model showed a significant but weak correlation between MIP and TFdi (*p* < 0.001, *R*^2^ = 0.43), Figure 3.

## Discussion

To our knowledge, this is the first study to address the coexistence of ICUAW and DW by using objective measurements in both cooperative and uncooperative patients. In our cohort, we found that these two entities do not always coexist and that ICUAW, but not DW, is independently related to both VFDs at 28 days and weaning success. Moreover, we found a significant but weak correlation between MIP and TFdi.

The occurrence of ICUAW was 80%, representing one of the highest percentages reported in the literature (21). However, we believe that the inclusion of uncooperative critically ill patients, the use of PENT (i.e., a screening tool for ICUAW) and the significant proportion of severely ill patients in our cohort of exclusively MV patients, may have contributed to the high occurrence of ICUAW. The occurrence of DW in our population was also found to be 80%, similar to what has been previously reported in the literature (22, 23).

The coexistence of ICUAW and DW was 65%, much higher than the 21% reported by Dres et al. (4). However, in their study, Dres et al. evaluated ICUAW only at the time of SBT and by using the MRCss; therefore, uncooperative patients with DW would not have been tested for ICUAW, potentially underestimating its real incidence. Interestingly, in a cohort of patients with established ICUAW, Jung et al. reported the coexistence of ICUAW and DW in 80% of the subjects; these results are similar to our findings when considering only the patients with ICUAW (74%; Table 2). Despite a higher coexistence in our cohort, we confirmed the lack of association between the ICUAW and DW (χ^2^ = 1.06, *p* = 0.304). Whether DW and ICUAW are manifestations of the same pathological process is currently a source of debate in the literature. Indeed, ICUAW and DW share similar risk factors and common pathophysiological mechanisms. However, from a histopathological point of view, limb muscle and diaphragm have different features, and limb muscle affected by ICUAW shows muscle necrosis more frequently than the diaphragm affected by DW (24). Our results seem to support the hypothesis that *ICUAW and DW are not necessarily expressions of the same biological phenomena* and that different factors may be involved in this disjunct development of weakness in critically ill patients.

In our cohort, patients without ICUAW had approximately 7 more FVDs (calculated at 28 days) than patients with ICUAW. Moreover, the development of ICUAW was associated with a significantly higher probability (OR 3.62) of a more challenging weaning process (i.e., difficult weaning, prolonged weaning, or no weaning vs short weaning). Although the relation between mechanical ventilation and diaphragm dysfunction (ventilator-induced diaphragmatic dysfunction) is well known (25), we found that DW was not related to either weaning success or the VFDs at 28 days. Different studies have reported the association between ICUAW and the duration of mechanical ventilation (6) and weaning failure (26, 27), but none of them has tested the DW coexistence.

When comparing our results with the largest published study on the coexistence of ICUAW and DW (5), we observed similar duration of mechanical ventilation in (1) patients ICUAW−/DW− (4 days), patients ICUAW+/DW+ (12 days), and patients ICUAW−/DW+ (7 days), Supplementary Table 1. Our data diverge from Dres et al. (5) for a higher duration of mechanical ventilation in patients ICUAW+/DW− (17 vs 7 days). Furthermore, and contrary to our results, Dres reported a higher impact of DW on weaning failure than did ICUAW. The important differences between the two studies could be explained by the fact that (1) we assessed the presence of DW and ICUAW throughout the entire ICU admission and not only at the time of liberation from MV; (2) by including uncooperative patients, we identified a large number of patients with ICUAW. Finally, we can’t exclude that in both DW− and DW+ the presence of an inappropriate diaphragm activity might have contributed to prolonging MV duration (28).

Despite the interesting findings, some limitations must be pointed out; firstly, we classified patients as suffering from ICUAW by using average bilateral PENT in uncooperative patients. PENT has been validated in ICU to diagnose critical illness myopathy and polyneuropathy (CIP) but it does not necessarily prove the presence of ICUAW. Notwithstanding, Hermans et al. demonstrated that an abnormal PENT, even in the absence of weakness, is independently associated with worse outcomes (29), including increased 5 years mortality (30). Secondly, concerning the diagnosis of DW, although MIP and TFdi have been both validated to diagnose respiratory muscle weakness, we did not use an objective maximal stimulation with BAMPS and we did not measure PdiTw to assess diaphragmatic strength (10). While TFdi describes the diaphragmatic function, MIP is a marker of global inspiratory strength (19, 22). Although the two techniques may not seem completely interchangeable, we found a strong correlation between MIP and TFdi with an increased dispersion for non-pathological values, Figure 2. In patients with pathological conditions (i.e., presence of DW) TFdi and MIP could be both useful as bedside screening tools for DW presence. To note, although MIP is easier to perform and has higher inter-rater reliability, it has not been tested against the gold standard (measures of PdiTw during BAMPS stimulations). Finally, the baseline characteristics of our population (a high SAPS II and prolonged mechanical ventilation) highly increased the chance of developing DW and/or ICUAW, explaining the low number of patients without weakness. Moreover, 62% of our patients suffered from CARDS, a risk factor for peripheral muscle weakness (31, 32), limiting the generalization of our results in classical ARDS.

In conclusion, this pilot study is the first to explore the coexistence of ICUAW and DW in both cooperative and uncooperative patients; a lack of association was found between DW and ICUAW when considering both cooperative and uncooperative patients. We found a strong correlation between ICUAW but not DW with the VFDs at 28 days and weaning success. A future larger study is warranted in order to confirm our results, and should also investigate the use of PdiTw measurement during BAMPS for the diagnosis of DW.

## Data Availability Statement

The raw data supporting the conclusions of this article will be made available by the authors, without undue reservation.

## Ethics Statement

The studies involving human participants were reviewed and approved by Comitato Etico di Brescia, Brescia, Italy. The patients/participants provided their written informed consent to participate in this study.

## Author Contributions

MiB, SP, and NL conceptualized the study. MiB, AB, RC, and FB collected the data. SP conducted the statistical analysis. MiB and SP drafted the manuscript. All authors critically revised the manuscript.

## Conflict of Interest

The authors declare that the research was conducted in the absence of any commercial or financial relationships that could be construed as a potential conflict of interest.

## Publisher’s Note

All claims expressed in this article are solely those of the authors and do not necessarily represent those of their affiliated organizations, or those of the publisher, the editors and the reviewers. Any product that may be evaluated in this article, or claim that may be made by its manufacturer, is not guaranteed or endorsed by the publisher.

## Abbreviations

ICUAW: intensive care unit-acquired weakness
DW: diaphragmatic weakness
MV: mechanical ventilation
MRCss: medical research council scale
BAMPS: bilateral magnetic phrenic nerve stimulation
TFdi: thickening fraction of the diaphragm
MIP: maximum inspiratory pressure
PENT: simplified peroneal nerve test
FRC: functional residual capacity
WIND: weaning according to a new definition
AMV: assisted mechanical ventilation
PSV: pressure support ventilation
SBT: spontaneous breathing trial
VFDs: ventilator-free days.
