# Supplementary material for: Occurrence and Effects on Weaning From Mechanical Ventilation of Intensive Care Unit Acquired and Diaphragm Weakness: A Pilot Study
Source: Front Med (Lausanne). 2022 Jul 22;9:930262. doi: 10.3389/fmed.2022.930262 (PMC9354572; doi:10.3389/fmed.2022.930262)

**Supplementary table 1.**  Demographics and outcomes characteristic in patients with and without Intensive Care Unit-Acquired Weakness (ICUAW).

|  | **No ICUAW**  **(N=16)** | **ICUAW**  **(N=57)** | **P-value** |
| --- | --- | --- | --- |
| **Presence of DW,** N°( %) | 11 (93%) | 48 (74%) | **0.173** |
| **Age (years),**Median [IQR] | 69 [11] | 64 [19] | 0.398 |
| **Body Mass Index (kg/m^2^),** Median [IQR] | 24.2 [6.1] | 27.8 [8.1] | 0.614 |
| **Admission diagnosis,**  N° (%) |  |  | 0.212* |
| Polytrauma | 1 (6%) | 3 (5%) | 0.341 |
| Respiratory Failure (NON-COVID) | 1 (6%) | 7 (12%) | 0.069 |
| Sepsis | 0 (0%) | 7 (12%) | 0.994 |
| Neurological Disease | 0 (0%) | 1 (1%) | 0.998 |
| CARDS | 13 (81%) | 32 (56%) | **0.006** |
| Cardiac Disease | 0 (0%) | 2 (3%) | 0.997 |
| Other | 1 (6%) | 1 (1%) | 0.999 |
| **Comorbidities,**  N° (%) |  |  | **0.042*** |
| 0 | 5 (31%) | 5 (8%) | 0.999 |
| 1 | 4 (25%) | 16 (28%) | **0.013** |
| 2 | 4 (25%) | 13 (22%) | **0.039** |
| ≥ 3 | 3 (18%) | 23 (40%) | **0.001** |
| **SAPS II,** Median [IQR] | 24 [10] | 33 [19] | **0.021** |
| **SOFA Score,** Median [IQR] | 4 [1] | 5 [5] | **0.032** |
| **MV duration (days),** Median [IQR] | 6 [3] | 12 [12] | **0.002** |
| **WIND,**  N° (%) |  |  | **0.011*** |
| Group NW: no separation attempt | 1 (6%) | 3 (5%) | 0.341 |
| Group 1:short weaning | 10 (62%) | 14 (24%) | **<0.001** |
| Group 2: difficult weaning | 4 (25%) | 15 (26%) | **0.059** |
| Group 3a: prolonged weaning | 0 (0%) | 15 (26%) | **<0.001** |
| Group 3b: weaning failure | 1 (6%) | 10 (17%) | **0.028** |
| **ICU LOS (days),** Median [IQR] | 8 [5] | 15 [11] | **0.002** |
| **Hospital LOS (days),** Median [IQR] | 27 [15] | 27.0 [14] | 0.275 |
| **Alive** (at Hospital discharge), N° (%) | 13 (81%) | 49 (86%) | 0.304 |

*List of abbreviations:*  MV duration=duration of mechanical ventilation; CARDS= Covid-19 ARDS; WIND (Weaning according to a New Definition); ICU LOS=ICU lenght of stay; Hospital LOS=Hospital lenght of stay; CI=95% Confidence Interval. *** p referred to the model considering the independent variable as numeric.**

**Supplementary table 2.** Demographics and outcomes characteristic in patients with and without Diaphragmatic weakness.

|  | **No DW**  **(N=14)** | **DW**  **(N=59)** | **P-value** |
| --- | --- | --- | --- |
| **Presence of ICUAW,** N° ( %) | 9 (64%) | 48 (81%) | **0.173** |
| **Age (years),** Median [IQR] | 71 [18] | 65 [14] | 0.736 |
| **Body Mass Index (kg/m^2^),** Median [IQR] | 25.6 [8.16] | 27.8 [7.62] | 0.227 |
| **Admission diagnosis,** N° (%) |  |  | 0.582 |
| Polytrauma | 1 (7%) | 3 (5%) | 0.341 |
| Respiratory Failure (NON-COVID) | 3 (21%) | 5 (8%) | 0.484 |
| Sepsis | 0 (0%) | 7 (11%) | 0.994 |
| Neurological Disease | 0 (0%) | 1 (1%) | 0.998 |
| CARDS | 7 (50%) | 38 (64%) | **<0.001** |
| Cardiac Disease | 0 (0%) | 2 (3%) | 0.997 |
| Other | 1 (7%) | 1 (1%) | 0.999 |
| **Comorbidities,** N° (%) |  |  | 0.521 |
| 0 | 4 (28%) | 6 (10%) | 0.530 |
| 1 | 2 (14%) | 18 (30%) | **0.003** |
| 2 | 3 (21%) | 14 (23%) | 0.050 |
| ≥ 3 | 5 (35%) | 21 (35%) | 0.206 |
| **SAPS II Score (value),** Median [IQR] | 33 [8] | 30 [19] | 0.706 |
| **SOFA Score,** Median [IQR] | 4 [1] | 4 [3] | 0.514 |
| **MV duration (days),** Median [IQR] | 10 [13] | 9 [10] | 0.994 |
| **WIND,** N° (%) |  |  | 0.806 |
| Group NW: no separation attempt | 1 (7%) | 3 (5%) | 0.341 |
| Group 1:short weaning | 4 (28%) | 20 (33%) | **0.003** |
| Group 2: difficult weaning | 5 (35%) | 14 (23%) | **0.048** |
| Group 3a: prolonged weaning | 2 (14%) | 13 (22%) | 0.014 |
| Group 3b: weaning failure | 2 (14%) | 9 (15%) | 0.054 |
| **ICU LOS (days),** Median [IQR] | 16 [14] | 13 [11] | 0.854 |
| **Hospital LOS (days),** Median [IQR] | 21 [19] | 27 [13] | 0.465 |
| **Alive** (at Hospital discharge), N° (%) | 11 (78%) | 51 (86%) | 0.621 |

*List of abbreviations:*  MV duration=duration of mechanical ventilation; CARDS= Covid-19 ARDS; WIND=Weaning according to a New Definition; ICU LOS=ICU lenght of stay; Hospital LOS=Hospital lenght of stay; CI=95% Confidence Interval.

**Supplementary Table 3: Unadjusted linear regression for mechanical ventilation duration.**

| ***Predictors*** | *Estimates* | *CI* | *p* |
| --- | --- | --- | --- |
| (Intercept) | 12.67 | 9.09 – 16.24 | **<0.001** |
| **Gender, Male (N°)** | 1.23 | -3.96 – 6.42 | 0.639 |
| **Presence of ICUAW (N°)** | -7.87 | -13.17 – -2.57 | **0.004** |
| **Presence of DW (N°)** | 3.66 | -2.36 – 9.68 | 0.229 |
| **Age (years)** | -0.14 | -0.33 – 0.04 | 0.129 |
| **Body Mass Index (kg/m^2^)** | -0.07 | -0.45 – 0.31 | 0.704 |
| **Comorbidities (N°)** | -1.71 | -3.87 – 0.45 | 0.119 |
| **SOFA Score (value)** | -0.77 | -1.53 – -0.00 | **0.049** |
| **SAPS II Score (value)** | -0.33 | -0.54 – -0.12 | **0.003** |

**Supplementary Table 4: Unadjusted linear regression for weaning from mechanical ventilation.**

| ***Predictors*** | *Odds Ratios* | *CI* | *p* |
| --- | --- | --- | --- |
| **Gender, Male (N°)** | 1.05 | 0.42 – 2.67 | 0.913 |
| **Presence of ICUAW (N°)** | 6.27 | 1.74 – 22.59 | **0.004** |
| **Presence of DW (N°)** | 1.04 | 0.35 – 3.14 | 0.937 |
| **Age (years)** | 1.02 | 0.10 – 10.38 | 0.157 |
| **Body Mass Index (kg/m^2^)** | 1.01 | 0.11 – 9.07 | 0.820 |
| **Comorbidities (N°)** | 1.19 | 0.31 – 4.61 | 0.386 |
| **SOFA Score (value)** | 1.24 | 0.39 – 4.00 | **0.002** |
| **SAPS II Score (value)** | 1.08 | 0.18 – 6.37 | **0.001** |

**Supplementary Figure 1:** Direct acyclic graphs (DAG) for counfounders selection. We hypothesized a direct correlation between ICUAW (or DW) and MV duration (or WIND), and we adjust (“cloding the back-doors”) for sex, age, SAPS IIand Bosy Mass Index.


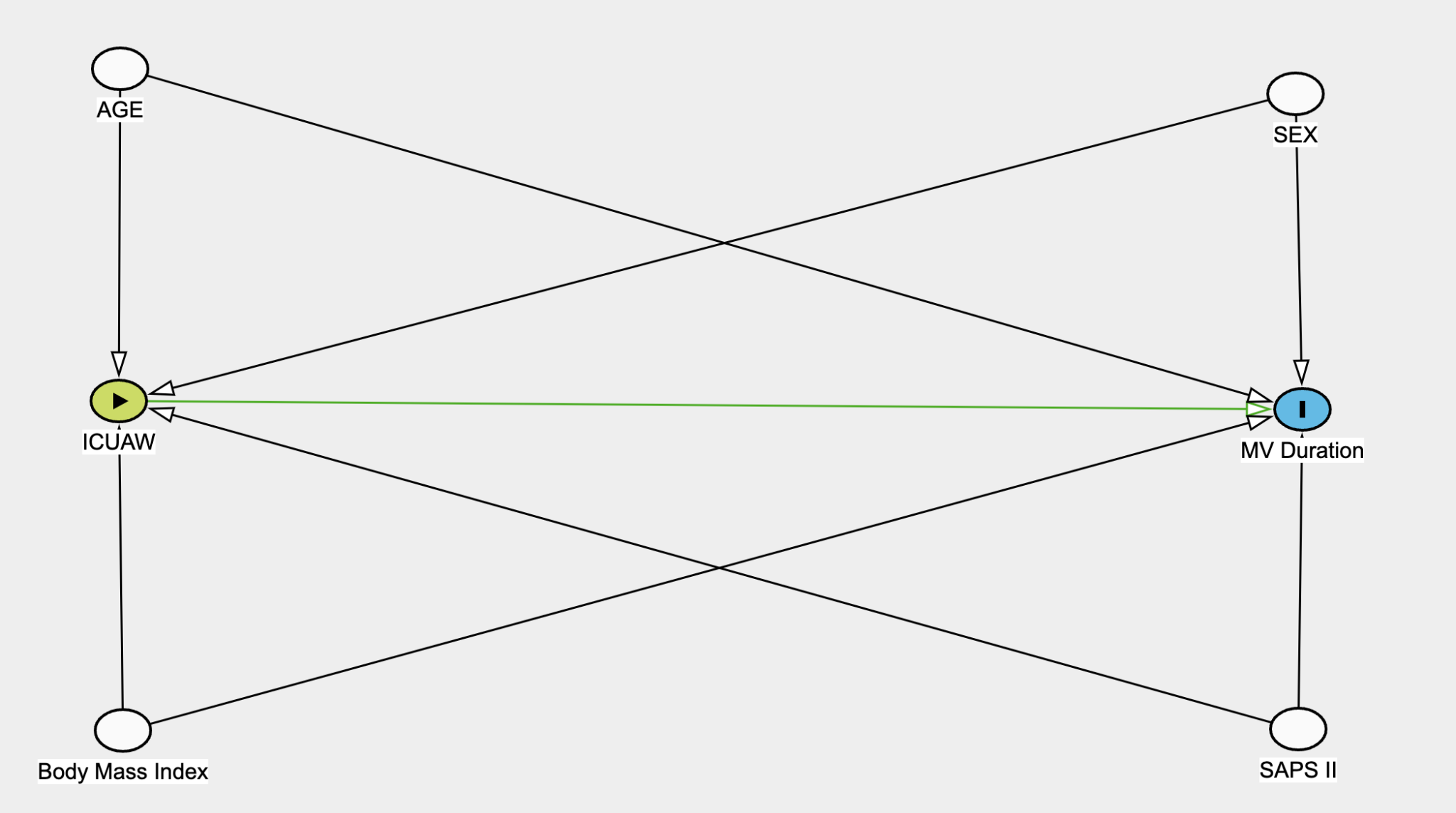

Supplement: Supplementary file 1 [file Data_Sheet_1.docx]
